# Plant derived substances with anti-cancer activity: from folklore to practice

**DOI:** 10.3389/fpls.2015.00799

**Published:** 2015-10-01

**Authors:** Marcelo Fridlender, Yoram Kapulnik, Hinanit Koltai

**Affiliations:** Institute of Plant Sciences, Agricultural Research Organization, Volcani Center, Bet Dagan, Israel

**Keywords:** plant, active compounds, anti-cancer agents, folklore, chemoprevention

## Abstract

Plants have had an essential role in the folklore of ancient cultures. In addition to the use as food and spices, plants have also been utilized as medicines for over 5000 years. It is estimated that 70–95% of the population in developing countries continues to use traditional medicines even today. A new trend, that involved the isolation of plant active compounds begun during the early nineteenth century. This trend led to the discovery of different active compounds that are derived from plants. In the last decades, more and more new materials derived from plants have been authorized and subscribed as medicines, including those with anti-cancer activity. Cancer is among the leading causes of morbidity and mortality worldwide. The number of new cases is expected to rise by about 70% over the next two decades. Thus, there is a real need for new efficient anti-cancer drugs with reduced side effects, and plants are a promising source for such entities. Here we focus on some plant-derived substances exhibiting anti-cancer and chemoprevention activity, their mode of action and bioavailability. These include paclitaxel, curcumin, and cannabinoids. In addition, development and use of their synthetic analogs, and those of strigolactones, are discussed. Also discussed are commercial considerations and future prospects for development of plant derived substances with anti-cancer activity.

## Introduction

Plants have had an essential role in the folklore of ancient cultures. In addition to the use as food and spices, plants have also been utilized as medicines for over 5000 years. Two remaining living traditions, the traditional Chinese medicine (TCM) and Ayurveda, the traditional Indian medicine (TIM) have provided most of the current knowledge related to medicinal plants (Goldman, 2001; Patwardhan et al., 2005). In TCM and TIM folklore, herbal medicines were prepared as teas, tinctures, poultices, powders, and other types of formulations (Balick and Cox, 1996; Samuelsson, 2004). The expertise to select the right plants, methods of drug concoction and their specific use has been first transferred orally from one generation to the next until set down (Kinghorn, 2001; Samuelsson, 2004).

It is estimated that 70–95% of the population in developing countries continues to use traditional medicines (Robinson and Zhang, 2011). Today medicinal herbs are defined as plants that contain valuable substances with therapeutic or beneficial effect in healing and prevention of various ailments in man and animals. Herbal products such as plant extracts, dry powders and parts of plants, fungi, and algae have been used as complementary treatments alongside conventional drugs (Li, 2002; Robinson and Zhang, 2011).

A new trend, that involved the isolation of plant active compounds begun during the early nineteenth century. This tendency led to the discovery of the analgesic (painkilling) drugs morphine and codeine from opium (*Papaver somniferum* L.); cocaine from *Erythroxylum coca*; the cardiac glycoside, digitoxin that was isolated from *Digitalis purpurea* and *Digitalis lanata* that has been used for cardiac conditions and as an anti-cancer drug, and quinine from *Cinchona calisaya* Wedd. and *Cinchona succirubra* Pav. ex Klotzsch that has antipyretic (fever reducing), antimalarial, analgesic, and anti-inflammatory properties. Some of these molecules are still in use (Newman et al., 2000; Kinghorn, 2001; Butler, 2004; Samuelsson, 2004; Balunas and Kinghorn, 2005; Elbaz et al., 2012; Menger et al., 2013). Such natural compounds provide a huge variety, often with strong biological activity and therefore play a significant role in the development of therapeutics treatments (Butler, 2004; Balunas and Kinghorn, 2005; Gordaliza, 2007).

Discovery of plant-derived substances has evolved during the last 200 years due to the variety of experience and expertise needed in order to identify such compound. Initially, a plant is identified by a botanist or ethnobotanist, ethnopharmacologist, or plant ecologist. Next, plant extracts followed by biological screening assays are performed by a phytochemist to identify the potential therapeutic activity followed by isolation of the active compound. Finally, molecular biology studies are required to reveal the mode of action and relevant molecular targets. The combination of these fields determines an interdisciplinary approach termed pharmacognosy (Kinghorn, 2001; Balunas and Kinghorn, 2005).

Today, it is estimated that about 25–28% of all modern medicines are directly or indirectly derived from higher plants demonstrating the enormous medicinal potential of plants that has been known for thousands of years in traditional medicine (Samuelsson, 2004; Chin et al., 2006). In the last decades, more and more new materials derived from plants have been authorized and subscribed as medicines (Balunas and Kinghorn, 2005; Chin et al., 2006). Several important examples for plant origin medicines are Arteether (Artemotil^®^), a sesquiterpene lactone isolated from *Artemisia annua* and serves for treatment of malaria (Van Agtmael et al., 1999; Graul, 2004), Galantamine (Reminyl^®^) an amaryllidaceae alkaloid from *Galanthus woronowii* used for Alzheimer treatment due to its activity as a selective acetylcholinesterase inhibitor (Heinrich and Teoh, 2004; Pirttilä et al., 2004), Apomorphine hydrochloride (Apokyn^®^) a dopamine receptor agonist produced in *Papaver somniferum* L. used to treat Parkinson’s disease (Deleu et al., 2004), Tiotropium bromide (Spiriva^®^) isolated from *Atropa belladonna* and used for to treat COPD (chronic obstructive pulmonary disease; Mundy and Kirkpatrick, 2004; Koumis and Samuel, 2005), Nitisinone (Orfadin^®^) a modified mesotrione from *Callistemon citrinus* that inhibits the 4-hydroxyphenylpyruvate dioxygenase (HPPD) enzyme and prevents accumulation of fumaryl and maleyl acetoacetate in the liver and kidneys (Hall et al., 2001; Mitchell et al., 2001).

Interestingly, many isolated substances against cancer are connected with interactions between plants and microbes. Such interactions are related to rhizospheric or endophytic bacteria, yeasts, and fungi. These microorganisms penetrate and reside within plants without injuring them or causing any disease. Furthermore, such microbes serve as a barrier for colonization by pathogenic microorganisms and participate in plant growth and plant defense response by production of a large variety of secondary metabolites (reviewed in Strobel and Daisy, 2003; Strobel et al., 2004; Ryan et al., 2008; Chandra, 2012; Kaul et al., 2012). Since almost all plants co-exist with at least one endophyte and many molecules have been isolated from such systems, this review does not aim to summarize this field and it should review somewhere else. Nonetheless, two recent studies revealed that extracts from *Chaetomium globosum* (Wang et al., 2012; Awad et al., 2014) and 5-Methyl phenazine-1-carboxylic acid that is produced by *Pseudomonas putida* (Kennedy et al., 2015) had cytotoxic effects against cancer cell lines. The similarity of secondary metabolites produce by endophytes and their hosts implies on gene transfer between them throughout co-evolution (Chandra, 2012). Taking advantage of biotechnology new drugs from endophytes can be manufactured in faster and controlled processes (Chandra, 2012; Kaul et al., 2012).

In addition to the drugs mentioned above, other plant-derived substances with anti-cancer activity such as Paclitaxel (Taxol^®^) and Camptothecin have been isolated and approved for use. Here we will focus on some plant-derived substances exhibiting anti-cancer activity, their mode of action and bioavailability. In addition, comparison of the use of natural vs. synthetic analogs as well as commercial consideration will be discussed.

## Examples for Plant-Derived Substances with Anti-Cancer Activity

Cancer is among the leading causes of morbidity and mortality worldwide. In 2012, 8.2 million cancer related deaths and approximately 14 million new cases were counted. The number of new cases is expected to rise by about 70% over the next two decades. Among men, the five most common sites of cancer diagnosed in 2012 were lung, prostate, colorectum, stomach, and liver cancer. Among women the five most common sites diagnosed were breast, colorectum, lung, cervix, and stomach cancer. It is expected that annual cancer cases will rise from 14 million in 2012 to 22 within the next two decades (http://www.who.int/mediacentre/factsheets/fs297/en/ and references within).

Today, solid tumors are surgically removed and patients receive adjuvant radiation treatment and chemotherapy that cause severe sides effects and dramatically reduce quality of life. In addition, the toxicity of some treatments restricts their use and effectiveness. Certain types of cancer such as breast cancer, can be treated using biological drugs (Herceptin), however the cost of these drugs is very high and their effectiveness is limited in most cases to certain kinds of tumors. In many cases, the tumor develops resistance to a particular drug and the patient is transferred to a different drug. In addition, many patients are treated with a combination of several drugs. More on cancer treatment and survivorship statistics may be found in DeSantis et al. (2014) and references within. Thus, there is no doubt that there is a real need for new efficient anti-cancer drugs with reduced side effects, and plants are a promising source for such entities. Due to the importance of fighting cancer and the variety of potential molecules offered by plants, over 60% of anti-cancer drugs are directly or indirectly derived from this kingdom (Gordaliza, 2007).

Probably the most well-known plant-derived anti-cancer drug is Paclitaxel (Taxol^®^). The cytotoxic activity of this taxane dipertene found in extracts from the bark of *Taxus brevifolia* Nutt. (Western yew) was first reported by Wani et al. (1971). Later on, other *Taxus* species were found to produce this molecule. Interestingly, In 1993, Taxol was also found to be produced at low levels by *Taxus*’ endophytic fungus *Taxomyces andreanae* (Stierle et al., 1993) and later on by other endophytic fungi (Guo et al., 2006) allowing its possible production by future microorganism fermentation. Taxol’s molecular structure is based on the A, B, and C rings that harbor two hydroxyl groups, two acetyl groups, one benzoyl group and an oxetane ring. The side chain A ring together with the benzoyl group at C2 and the oxetane ring are responsible for Taxol’s anti-cancer activity. The C3 amide-acyl group in the C12 chain maintains this activity and the hydroxyl group at C2 enhances it (Kingston, 2000, 2001; Malik et al., 2011).

As other plant-derived compound such as vinca alkaloids, Taxol disrupts microtubule function. However, in contrast to vinca alkaloids that disrupt microtubule assembly by binding depolymerized microtubules, Taxol (essentially all taxanes) inhibits microtubule disassembly by binding the polymerized microtubules (Xiao et al., 2006; Hait et al., 2007; Prota et al., 2013). Microtubules are composed of αβ-tubulin heterodimers and are highly dynamic polymers. Taxol stabilizes this dynamicity by binding to and altering the conformation of the microtubules. Taxol was also found to reduce the binding of microtubule-associated proteins (MAPs). Despite the fact that MAP binding induces conformational rearrangements of α- and β-tubulin that promote an overall stabilization of microtubules, Taxol binding to the MAP-microtubule complex leads to further stabilization of this complex (Xiao et al., 2012). These disturbances in microtubule lead to prevention of normal mitotic spindle formation and function resulting in prevention of mitosis and thus cell proliferation (Bharadwaj and Yu, 2004; Hait et al., 2007; Ganguly et al., 2010; Malik et al., 2011; Priyadarshini and Aparajitha, 2012).

A common problem observed in Taxol-treated patients is development of drug-resistance over time. However, a combined treatment of Taxol together with down-regulation of expression of Bcl-2 (B-cell lymphoma 2), a cell death regulator, or other apoptotic related genes inhibits invasion, angiogenesis tumor growth, and maintains Taxol sensitivity in different types of cancer cells (George et al., 2009; Yang et al., 2010; Zhou et al., 2010; Maraz et al., 2011; Sun et al., 2011; Korbakis and Scorilas, 2012; Shajahan et al., 2012; Morales-Cano et al., 2013).

Although discovered in the early 1970s of the twentieth century, it took over 25 years to bring Taxol to the market. It was approved by the Food and Drug Administration (FDA) only in 1992 for the treatment of metastatic ovarian cancer. Clinical trials also demonstrated encouraging results for other cancer types such a head, neck, lung, and breast cancer (Suffness, 1995; Gordaliza, 2007). This anti-cancer drug, that is now produced in a semi-synthetically (see below) process reached the list of the top-selling 20 drugs and sold above $1 billion yearly in 1999 (Kingston, 2000; Kinghorn, 2001; Malik et al., 2011) with a peak of $1.6 billion in 2000 before generic drugs appeared in the market (Dwivedi et al., 2010; Buchwald-Werner and Bischoff, 2011). In 2002, sales for Taxol and Camptothecin, a DNA Topoisomerase I inhibitor, were more than $2.75 billion, approximately a third of the anti-cancer drug market. Docetaxel (Taxotere), Taxol’s analog, had sales of $3 billion in 2009 (Demain and Vaishnav, 2011). Taxol’s success serves as an example and encouraging story for discovery and bringing to market additional plant-derived substances.

Chemoprevention represents a different attitude in the battle against cancer. This approach was defined by (Sporn et al., 1976; Sharma et al., 2005; Johnson and Mukhtar, 2007) as ingestion of dietary or pharmaceutical compounds in order to prevent or delay carcinogenic processes. Many potential chemopreventive secondary metabolites in both plant extracts as well as purified molecules isolated from teas, herbs, spices, fruits and vegetables have been explored (Kelloff et al., 1994; Kinghorn, 2001).

Curcumin (diferuloylmethane) is the most prominent chemopreventive agent studied. This yellow–orange turmeric powder, is a polyphenol that accumulates in the rhizome of *Curcuma longa*. Both TCM and TIM have used curcumin as a medicine for treatments of diseases (Aggarwal et al., 2003; Sharma et al., 2005; Jackson et al., 2013; Thangapazham et al., 2013). Due to its capabilities to regulated important transcription factors, cytokines, protein kinases, adhesion molecules and redox status, curcumin can serve as an anti-inflammatory, anti-oxidant, anti-proliferative, anti-angiogenic, and antineoplastic agent. Thus it has been used to treat many different conditions such as neurodegenerative diseases, cardiovascular diseases, diabetes, allergy, asthma and bronchitis, inflammatory bowel diseases, rheumatoid arthritis, renal ischemia, psoriasis, scleroderma, acquired immunodeficiency disease (AIDS), and cancer as well as an anti-aging and scar formation agent (Aggarwal et al., 2003; Anand et al., 2007; Gordaliza, 2007; Johnson and Mukhtar, 2007; Aggarwal and Harikumar, 2009; Thangapazham et al., 2013).

Hundreds of studies on curcumin anti-cancer activities were published and are discussed here only briefly. Both *in vitro* and *in vivo* assays have shown inhibition of cell growth in many types of cancerous cells (Anand et al., 2008; Kunnumakkara et al., 2008). Maybe one of the most important features related to curcumin is the suppression of the transcription factor NF-κ-B, a central protein in many types of cancer (Marín et al., 2007). This inhibition causes reduction in expression of NF-κ-B target genes such as *COX-2* and *cyclin D1* resulting in apoptosis (Aggarwal et al., 2003; Thangapazham et al., 2013). Induction of apoptosis by curcumin was also shown recently when this substance was given together with the estrogen receptor antagonist Tamoxifen for treatment of melanoma. Interestingly, also autophagy was induced using such combination (Cao et al., 2007; Chatterjee and Pandey, 2011).

Curcumin can act also as chemopreventive agent of photocarcinogenesis by protecting the skin through diminishing oxidative stress and suppressing inflammation (Aggarwal et al., 2003; Heng, 2010). As Taxol, curcumin has also been shown to affect microtubule assembly leading cells to mitotic arrest (Gupta et al., 2006). Recently, this type of action by curcumin was found non-specific only to cancer cells implying its possible impact also on normal cells (Jackson et al., 2013). Therefore, additional studies to assure this plant-derived substance influence on normal cells and its used in combination with other anti-cancer drugs are required.

*Cannabis sativa* has also been used in the TCM, mainly to treat malaria, constipation, rheumatic pains and during childbirth. It was only in the nineteenth century that an Irish scientist and physician, O’Shaughnessy had found that in India it is used as an analgesic, anticonvulsant, antispasmodic, anti-emetic, and hypnotic agent. These observations caused its spreading into Europe and US, until it was outlawed in 1928 and 1937, respectively due to its negative effects (reviewed in Robson, 2001).

The plant *Cannabis sativa* is known to contain more than 60 terpenophenolic compounds, named phytocannabinoids (Mechoulam et al., 2002; Russo and Guy, 2006; Alexander et al., 2008; Pertwee, 2008). The first active compound isolated from *Cannabis sativa* was Δ^9^-tetrahydrocannabinol (Δ^9^-THC), the active component of marijuana (Gaoni and Mechoulam, 1964). Cannabinoids are mainly used in the last two centuries as supporting drugs for patients that receive either radiation or chemotherapies. These drugs ease common symptom and side effects related to such treatments as nausea, vomiting, cachexia, and loss of appetite (Robson, 2001; Tramèr et al., 2001; Hall et al., 2005; Massa et al., 2005; Massa and Monory, 2006; Grotenhermen and Müller-Vahl, 2012).

Both natural and synthetic cannabinoids act by interacting with two specific G-protein-coupled cannabinoid receptors, known as cannabinoid type 1 receptor (CB_1_ receptor) and cannabinoid type 2 receptor (CB_2_ receptor). The role of CB_1_ receptors in regulation of neurotransmitter release and its involvement in cannabinoid psychoactive effects is well known. In accordance, these receptors are positioned in central and peripheral nerves (Massa and Monory, 2006; Matias and Di Marzo, 2006; Howlett et al., 2010; Pertwee et al., 2010; Smith et al., 2010; Stella, 2010; Turu and Hunyady, 2010). On the hand, the function of CB_2_ receptors which are most abundant in the immune system remained vague for many years although there were evidences for their participation in regulation of cytokine’s function and release (Massa and Monory, 2006; Matias and Di Marzo, 2006). Today there is enough data indicating CB_2_ role’s in a variety of immunological functional responses mainly through progression of inflammation events. Participation of CB_2_ in different signal transduction pathways implies its essential function in maintenance of a homeostatic immune balance (Cabral and Griffin-Thomas, 2009; Mukhopadhyay et al., 2010; Roche and Finn, 2010). In addition, in several different studies, expression of CB_2_ at different levels has been also detected in the central nervous system and brain (Onaivi, 2011). Moreover, some studies indicate this receptor can be used as a selective molecular target for therapeutic treatments of both inflammation-related conditions and neuropathic diseases that display hyper-inflammation processes (Cabral and Griffin-Thomas, 2009; Atwood and Mackie, 2010; Cencioni et al., 2010; García-Gutiérrez et al., 2010; Atwood et al., 2012).

The discovery of Δ^9^-THC receptors, led to the assumption that endogenous ligands similar to this molecule should exits. Indeed, two of these ligands, anandamide and 2-arachidonoyl glycerol (2-AG), were exposed shortly after. This was followed by identification of other endogenous ligands as well as synthetic pathways responsible for both synthesis and degradation of these cannabinoids (Kogan and Mechoulam, 2006; Massa and Monory, 2006; Cabral and Griffin-Thomas, 2009; Atwood and Mackie, 2010; Pertwee et al., 2010; Stella, 2010; Onaivi, 2011).

The use of cannabinoids as anti-cancer agents is still under debate due to both cancer promoting and inhibiting effects shown in the last centuries (Hall et al., 2005; Massa et al., 2005; Velasco et al., 2015). The fact that cannabinoids play a role in cell fate decision, proliferation, and apoptosis (Guzmán et al., 2001) might imply different effects under different conditions Ligresti et al. (2003) demonstrated that the endocannabinoid system may play a role in cancer differentiation (by decreasing the levels of endogenous agonists in differentiated cells vs. undifferentiated ones), cell growth and cell migration leading to metastases. On the other hand, their results imply that in the gastrointestinal system cannabinoid receptors are involved in inhibition of cell proliferation of colorectal carcinoma (Ligresti et al., 2003; Massa and Monory, 2006). In studies using cell lines, the antineoplastic effect of both natural and synthetic cannabinoids, cannabinoids agonist, and endocannabinoids have been shown for several cancer types including carcinomas (skin, lung, prostate, and uterine), neuroblastoma, gliomas, lymphomas, thyroid epithelioma, and breast cancer. Although the mode of action leading to these effect is not completely clear, cannabinoids receptors appear to mediate it (reviewed in Hall et al., 2005; Oesch and Gertsch, 2009; Sharma et al., 2014; Velasco et al., 2015). In contrast to these *in vitro* studies, long-term exposure to cannabis carcinogens during smoking may lead to cancer in the aero-digestive tract and lungs (Hall et al., 2005; Aldington et al., 2008), suggesting that cannabinoids should be administrated with caution.

## Development and Use of Synthetic Analogs to Plant Derived Substances

One limiting trait related to many plant secondary metabolites is their poor solubility or poor bioavailability that delays manufacturing of large amounts required to serve as medicines (Lipinski et al., 1997). The main solution adopted for different plant derived substances is the use of semi-synthetic or synthetic analogs. One known example is morphine that has been modified (to morphine-6-glucuronide) in order to obtain better therapeutics features such as side effect (Lötsch and Geisslinger, 2001).

In the case of Taxol, in addition to the low amounts produced in all *Taxus* species (Castor and Tyler, 1993; Kingston, 2001; Parc et al., 2002; Yazdani et al., 2005) this compound is insoluble in water (Kingston, 2000, 2001). Several different approaches were explored in attempts to obtained higher amounts of Taxol. At the end of the twentieth century, two groups reported that needles of the European *Taxus*, *Taxus baccata* L., contain a high concentration of two Taxol precursors, 10-deacetylbaccatin III and baccatin (Denis et al., 1988; Guenard et al., 1993). In addition, plant tissues culture and several semisynthetic approaches were tested in order to produce higher amounts of Taxol. The main step forward was achieved by combining the use of 10-deacetylbaccatin III and a semisynthetic process for production of the drug. Today, Taxol and its semisynthetic soluble analog Docetaxel (Taxotere™) are manufactured in a multi-step semisynthetic process, however additional improvements are needed in order to reach future market demands of this important drug (Kingston, 2001; Parc et al., 2002; Guo et al., 2006; Malik et al., 2011).

Similarly to Taxol, also curcumin is insoluble in water. In addition, due to reduced absorption in liver and in intestinal walls and systemic elimination, curcumin has poor bioavailability (Aggarwal et al., 2003; Sharma et al., 2005) that has been mainly related to rapid metabolism, fast systemic elimination and poor absorption (Anand et al., 2007). Therefore, curcumin analogs have been prepared and their capability to retain therapeutic activities has been tested. One of the main modifications applied was the change of the beta-diketone in curcumin to a monocarbonyl dienone. These analogs demonstrated good bioavailability and therapeutic effect in rodents (Gordaliza, 2007; Mosley et al., 2007). At least two other approaches to increase curcumin’s bioavailability are being explored. The first approach takes advantage of adjuvants given with curcumin, that will block its metabolic pathways and/or increase its permeability resulting in higher blood concentrations and increase in its bioavailability (Anand et al., 2007; Basnet and Skalko-Basnet, 2011), while the second technique deals with new formulations of curcumin using nanoparticles (Anand et al., 2010; Basniwal et al., 2011; Chang et al., 2013), liposomes (Sun et al., 2010; Dhule et al., 2012), micelles (Hong et al., 2011; Song et al., 2011; Yang et al., 2012), and phospholipid complexes (Ghosh et al., 2011; Gupta and Dixit, 2011).

Modifications, formulations and synthetic compounds were also developed for the *Cannabis* derived cannabinoid Δ^9^-THC (Kogan and Mechoulam, 2006). One example is cannabidiol (CBD) another natural cannabinoid from *Cannabis sativa* and the major component of Sativex, a drug for multiple sclerosis and cancer pain. This cannabinoid has low affinity to CB_1_ and CB_2_ and therefore has reduced psychotropic effects (McPartland et al., 2007; Capasso et al., 2008) and it is prescribed in several European countries. Synthetic capsules of a different drug, Dronabinol, with Δ^9^-tetrahydrocannabinol as its main compound has been available in the US since 1985, while Nabilone that is a THC synthetic analog became available in 1983. Today, these drugs are also available in other countries (Robson, 2001; Tramèr et al., 2001; Howlett et al., 2010; Grotenhermen and Müller-Vahl, 2012).

Moreover, the endocannabinoid system is highly selective and has temporal specificity of activation. In the central nerve system they are not stored in vesicles but release on-demand from membrane precursors. In non-neuronal organs their expression is spatial and limited and condition-specific (Massa et al., 2005; Massa and Monory, 2006). For affecting different organs and have different activities development of different analogs and formulation for different conditions might be required.

Strigolactones (SLs) are a new potent group of plant hormones that might serve as anti-cancer drugs. These plant hormones were first identified about 50 years ago as germination stimulants of the parasitic plants *Striga* and *Orobanche* (Cook et al., 1966; reviewed in Xie et al., 2010). This family of terpenoid lactones is synthesized through the carotenoid synthesis pathway. Their molecular backbone is built from an ABC ring system that is connected to a butenolide through an enol ether bridge (Matusova et al., 2005; Xie et al., 2010). Moreover, SLs act in other developmental processes across the plants such as suppressors of outgrowth of pre-formed axillary shoot buds (Gomez-Roldan et al., 2008; Umehara et al., 2008), and regulators of root development (Kapulnik et al., 2011; Ruyter-Spira et al., 2011). Different plants produce different SLs and so far approximately 15 of these hormones have been structurally characterized. Further, synthetic analogs have been produced in different laboratories (reviewed by Koltai and Prandi, 2014).

Pollock et al. (2012) were the first to demonstrate the effect of SLs on cancer cells. Six SLs analogs were shown to inhibit growth and survival of breast cancer cell lines. All six analogs induced G2/M arrest but differ in extent of apoptosis. Growth inhibition in normal cells treated with these analogs was observed only with the highest SL concentration used, suggesting a dose response to these molecules. In a subsequent study (Pollock et al., 2014), inhibition was observed in both solid and non-solid cancer cells such as colon, lung, prostate, melanoma, osteosarcoma, and leukemic cell lines. Moreover, SLs were shown to act as new anti-cancer agents in inhibition of breast cancer in xenograft model with low toxicity (Mayzlish-Gati et al., 2015). Although the complete mode of action has not been revealed yet, it was shown that the tested cancer cells are arrested at G2 through activation of the p38 and JNK1/2 MAPKs causing stress induction (Pollock et al., 2012, 2014). In addition, SLs affect the integrity of the microtubule network and therefore may inhibit the migratory phenotype of the highly invasive breast cancer cell lines that were examined (Mayzlish-Gati et al., 2015).

These results suggest that SLs are potent and promising anti-cancer agents that induce cell cycle arrest, cellular stress and apoptosis in cancer cells. Furthermore, the variety of SLs available in nature from different plants enables development of different analogs for a large range of cancer treatments and possibly other pathological conditions and/or personalized treatments.

## Commercial Considerations and Future Prospects

The traditional belief that a single drug, “silver bullet” is sufficient to treat a single disease has been questioned. This belief relies on the premise that human diseases have a uniform underlying genetic basis across patient’s populations. However, recent advances in genomics demonstrate genetic diversity, i.e., polymorphism, implying that different patient populations may require different tailored drugs to their treatment, as personalized medicine. Moreover, the shortage in successful new chemical entities together with a focus of the pharmaceutical industry on such molecules to serve as “silver bullets” and the shift of companies from unpredictable research to a more steady businesses and revenues had created a crisis and loss of faith of public opinion in “silver bullets.”

The use of herbal medicines offers a way to alleviate this crisis in drug development. There are three main advances for herbal medicine: (1) utilizing the traditional herbal medicine knowledge may give rise to an inexpensive and more rapid discovery of new drugs; (2) herbal remedies offer a holistic approach that complements the disease targeted approach of “Silver bullets”; (3) synergy between the various components of the herbs which are an important element of their overall medical effects (Li, 2002).

The main disadvantage related to herbal medicines is the lack of international standardization in terms of methods for evaluating their composition, efficacy, safety, and quality, consistent manufacturing practices, regulation and approval processes. Ironically, vast knowledge and experience in drug development is available in the pharmaceutical industry. Therefore, combining the benefits provided by both traditional and modern medicine has been previously suggested as a promising approach in order to reveal and bring to market new plant-derived substances. However, in the last centuries only several herbal medicines or botanical drugs have been approved by health authorities for human use (Calixto, 2000; Li, 2002; Liu and Wang, 2008; Schmidt et al., 2008; Patwardhan and Mashelkar, 2009; Graziose et al., 2010; Sahoo et al., 2010; Choudhary and Sekhon, 2011; Newman and Cragg, 2012). Collaboration and coordination between World Health Organization (WHO), Federal Drug Administration (FDA), European and other regulatory agencies, and the pharmaceutical industry worldwide may lead to clear guidance for development of herbal medications while taking advantage of the huge potential held by traditional medicine for development of both anti-cancer and other health promoting drugs.

### Conflict of Interest Statement

The authors declare that the research was conducted in the absence of any commercial or financial relationships that could be construed as a potential conflict of interest.
